# Meningitis in a School-Aged Child due to *Haemophilus influenzae* Type E during the Post-Conjugate Vaccine Era—Monroe County, NY, 2011

**DOI:** 10.3390/vaccines2010107

**Published:** 2014-02-13

**Authors:** Byron S. Kennedy, Anita C. Weimer, Brenden Bedard, Jennifer L. Nayak, Drew Sacheli, John Ricci, Donna D. Meyer, Donna Hubbard

**Affiliations:** 1 Monroe County Department of Public Health, 111 Westfall Rd., Rochester, NY 14620, USA; E-Mails: acweimer@monroecounty.gov (A.C.W.); bbedard@monroecounty.gov (B.B.); dsacheli@monroecounty.gov (D.S.); jricci@monroecounty.gov (J.R.); dhubbard@monreocounty.gov (D.H.); 2 Department of Pediatrics, University of Rochester Medical Center School of Medicine and Dentistry, 601 Elmwood Ave, Box 690, Rochester, NY 14620, USA; E-Mail: jennifer_nayak@urmc.rochester.edu; 3 Mendon Pediatrics, 30 Assembly Drive, Suite 101 PO Box 488 Mendon, NY 14506, USA; E-Mail: drmeyer@mendonpediatrics.com

**Keywords:** *Haemophilus influenza*, public health, real-time polymerase chain reaction (RT-PCR)

## Abstract

In late October 2011, the Monroe County Department of Public Health (MCDPH) was notified of a suspected case of meningitis in a 9-year old girl from Monroe County, NY. Laboratory testing at the New York State Department of Health (NYSDOH) Wadsworth Center confirmed the identification of *Haemophilus influenzae* serotype e (Hie) isolated from the patient’s cerebrospinal fluid (CSF) using real-time polymerase chain reaction (RT-PCR). The universal immunization of infants with conjugate *H. influenzae* type b (Hib) vaccine has significantly reduced the incidence of invasive Hib disease, including meningitis, one of the most serious complications for infected children. Not surprisingly, as the epidemiology of invasive *H. influenzae* continues to change, non-Hib serotypes will likely become more common. The findings reported here underscore the importance for clinicians, public health officials, and laboratory staff to consider non-Hib pathogens in pediatric cases of meningitis, especially when initial investigations are inconclusive.

## 1. Introduction

During the pre-*Haemophilus influenzae* serotype b (Hib) conjugate vaccine era, Hib was the cause of more than 95% of invasive *H. influenzae* disease among younger children [1]. Further, meningitis occurred in about two-thirds of these children with invasive Hib disease, with 15%–30% of survivors having serious neurological sequelae such as hearing impairment, mental retardation, seizure disorder, cognitive and developmental delay, and paralysis. 

The Center for Disease Control and Prevention Emerging Infection Program’s Active Bacterial Core Surveillance (ABCs) system suggests that Hib currently accounts for a rate of 0.20/100,000 cases among children <5 years based on provisional 2012 data [2]. The universal recommendation for the immunization of infants in the United States beginning at two months of age with conjugate *H. influenzae* type b (Hib) vaccine began in 1991 [3]. This has significantly reduced the incidence of invasive Hib disease, including meningitis, one of the most serious complications for infected children. As the epidemiology of invasive *H. influenzae* continues to change, non-Hib serotypes such as *Haemophilus influenzae* serotype e (Hie) are increasing. We present the case of a patient who was clinically diagnosed with bacterial meningitis but all routine laboratory cultures showed no growth at the local lab. We then discuss the importance of public health involvement when a bacterial meningitis case is identified but etiology of the organism is unknown. 

## 2. Case Report

On October 24, 2011 (Day #1), a previously healthy 9-year old girl with a 2-week history of upper respiratory tract infection symptoms, including a nonproductive cough and negative strep throat culture, presented to a pediatric office with fever (Tmax of 103 degrees F), headache, stiff neck, irritability, and no rash. She was presumptively diagnosed with bacterial meningitis and given a single dose of intramuscular ceftriaxone (50 mg/kg) before being transferred to a local emergency department. At the hospital, cerebrospinal fluid (CSF) obtained through lumbar puncture revealed a cloudy appearance, with a white blood cell (WBC) count of 2,942/mm^3^ (polys 88%), glucose of 5 mg/dL, and protein of 196 mg/dL; gram stain initially showed no organisms except, on cytospin, four gram-negative bacilli (not coccobacilli) were noted; cultures were negative. A head computed tomography (CT) scan with contrast suggested acute on chronic pansinusitis; subsequent magnetic resonance imaging (MRI) of the brain with contrast also demonstrated pansinusitis with no evidence of intracranial abscess or tracking from the sinuses. The patient was started on intravenous ceftriaxone, gentamycin, and vancomycin; no steroids were administered, given the unclear gram stain results and low suspicion for Hib meningitis. The patient was transferred to the pediatric intensive care unit (PICU). Since meningococcal disease could not be ruled out, the local health department was contacted to initiate an investigation. The patient significantly improved and was transferred from the PICU to a regular pediatric floor on October 26 (Day #3); at that time, her antibiotic regimen was switched to ceftriaxone alone as the initial negative CSF culture following only a single dose of ceftriaxone suggested that the organism was susceptible to that antibiotic. On October 30 (Day #7), she was discharged to home. On follow up with her pediatrician on November 2 (Day #10), she reported some mild headaches and fatigue but, otherwise, had no neurological deficits and was doing well. She completed a 14-day course of ceftriaxone.

On October 26, 2011 (Day #3), Monroe County Department of Public Health (MCDPH) staff interviewed the patient’s mother to determine potential exposure and need for post exposure prophylaxis (PEP) for bacterial meningitis due to invasive *H. influenzae* and/or meningococcal disease. The patient’s family received prescriptions for PEP: ciprofloxacin for the parents and rifampin for the younger sibling. The New York State Immunization Information System (NYSIIS) confirmed that the patient and the younger sibling were up to date in their immunizations, including the Hib conjugate vaccine (with doses given at 2, 4, 6 and 15 months old). On October 27, 2011 (Day #4), laboratory testing at the New York State Department of Health Wadsworth Center identified Hie from the patient’s CSF using RT-PCR. No further action was taken by MCDPH staff regarding potential PEP.

## 3. Conclusions

In the post-Hib conjugated vaccine era, Waggoner-Fountain *et al*. first reported on four cases of non-type b encapsulated *H. influenzae* meningitis diagnosed in children here in the United States (two due to Hie and two due to *H. influenzae* type f [Hif]) [4]. Among these cases, only one child was school-aged: a 6-year old boy. Despite his age, he had only received a single dose of Hib conjugated vaccine at 15 months old. The boy’s CSF revealed a WBC count of 3,440/mm^3^, glucose of 34 mg/dL, and protein of 80 mg/dL. Laboratory testing identified Hie in his blood and CSF; he was treated with ceftriaxone for ten days and fully recovered. 

For the post-Hib conjugated vaccine era, we are not aware of any other published cases of meningitis due to Hie among previously healthy school-aged children here in the U.S.; however, Ladhani *et al*. reported Hie meningitis amongst two previously healthy children in England and Wales [5]. The identification of Hie is likely influenced by the serotyping method used, as Satola *et al*. have shown that PCR is the gold standard for capsule typing [6]. Indeed, slide agglutination serotyping (SAST) may be unreliable and, in particular, may overestimate the frequency of *H. influenzae* type a (Hia). This finding may account for the relatively high percentage of Hia noted among children with invasive disease in one study [7]. 

As surveillance for *H. influenzae* has improved during the post-Hib conjugate vaccine era, including referral of isolates for serotyping and wider use of PCR, research has shown that serotype e is increasing [5,8]. This case report underscores the importance of using real-time PCR as a rapid and accurate test to diagnose acute bacterial meningitis when cultures are negative. The changing epidemiology of invasive *H. influenzae* disease underscores the success of the Hib conjugate vaccine; it also suggests there might be a need to develop vaccines that cover other serotypes in the future.

For invasive *H. influenzae*, current guidelines specify chemoprophylaxis with a four-day course of rifampin for household contacts of cases involving Hib but no other serotypes [9]. At this time, it is not clear what the efficacy is for chemoprophylaxis in non-b typeable disease, such as Hie [10].

Indeed, knowing the risk of secondary transmission from invasive non-Hib index cases will require more data from continued surveillance efforts. 
